# Genetic Modulation of Rpd3 Expression Impairs Long-Term Courtship Memory in Drosophila

**DOI:** 10.1371/journal.pone.0029171

**Published:** 2011-12-15

**Authors:** Helen L. Fitzsimons, Maxwell J. Scott

**Affiliations:** 1 Institute of Molecular BioSciences, Massey University, Palmerston North, New Zealand; 2 Department of Genetics, North Carolina State University, Raleigh, North Carolina, United States of America; Alexander Flemming Biomedical Sciences Research Center, Greece

## Abstract

There is increasing evidence that regulation of local chromatin structure is a critical mechanism underlying the consolidation of long-term memory (LTM), however considerably less is understood about the specific mechanisms by which these epigenetic effects are mediated. Furthermore, the importance of histone acetylation in Drosophila memory has not been reported. The histone deacetylase (HDAC) Rpd3 is abundant in the adult fly brain, suggesting a post-mitotic function. Here, we investigated the role of Rpd3 in long-term courtship memory in Drosophila. We found that while modulation of Rpd3 levels predominantly in the adult mushroom body had no observed impact on immediate recall or one-hour memory, 24-hour LTM was severely impaired. Surprisingly, both overexpression as well as RNAi-mediated knockdown of Rpd3 resulted in impairment of long-term courtship memory, suggesting that the dose of Rpd3 is critical for normal LTM.

## Introduction

Much effort has been focused over the last four decades on teasing apart the molecular mechanisms that underlie the long-lasting synaptic growth associated with consolidation of LTM. Recent evidence indicates that alterations in local chromatin structure mediated through modification to histones play a critical role in the long-term regulation of plasticity-related genes [1], [2], [3].

Increased acetylation of specific lysine residues on the N-terminals of histone tails relaxes the chromatin structure, allowing access of transcriptional machinery to promote gene expression, as well as providing docking sites for transcription factors. The state of acetylation of a particular lysine residue is governed by a balance between the activities of histone acetyltransferases (HATs) and HDACs [4]. In mature cells, the tissue-specific patterns of gene expression are maintained, in part, via control of histone acetylation and it was previously believed that after development these patterns remained static, however it is now clear that that activity-dependent changes in histone acetylation occur in post-mitotic neurons [3], [5], [6]. Transcription is an essential requirement for LTM and increased histone acetylation correlates with formation of memory in several animal models of LTM [3], [7].

Deficits in LTM are associated with decreased histone acetylation. CREB binding protein (CBP) is a HAT that acetylates histone H3 and interacts with CREB, a key transcription factor in formation of LTM. CBP^+/−^ mice display deficits in associative memory [1], as do mice with focal knockouts of CBP in the hippocampus [8]. Further, disruption of HAT activity via a dominant negative form of CBP also impairs spatial memory [9]. Conversely, inhibition of histone deacetylation correlates with improvements in long-term memory. Application of HDAC inhibitors sodium butyrate (NaBu), trichostatin A or suberoylanilide hydroxamic acid results in improvement in performance in several rodent memory tests [7], [10], [11]. Further, in mice trained to a subthreshold level, a transient memory can be transformed into a long-lasting stable memory on administration of NaBu immediately after training [12]. Overexpression of HDAC2, but not HDAC1, in the mouse brain impairs LTM, whereas knockout of HDAC2 results in enhancement of both STM and LTM [13]. In another study, Cre-recombinase mediated knockout of HDAC3 in the CA1 of the adult mouse hippocampus was also shown to enhance LTM, with no effect on STM, demonstrating that HDAC3 is also a key negative regulator of LTM [14]. These data contribute to a growing body of evidence that LTM is regulated by modulation of histone acetylation at specific genes, however the precise mechanisms by which specific HDACs control LTM requires further investigation.

Drosophila is a desirable model for molecular dissection of memory due to its reproducible memory assays and its tractability to genetic analysis. In particular for memory research, the ability to restrict gene expression to subregions of the brain with GAL4 drivers [15], [16] combined with temporal modulation of gene expression using the GAL80ts or Geneswitch systems [15], [17] provides a favorable advantage over other model system.

We have a particular interest in the role of histone acetylation in Drosophila courtship memory. Courtship in Drosophila requires multimodal sensory input, involving the chemosensory, mechanosensory, visual and olfactory pathways [18]. In males, this manifests as the display of several stereotypic behaviors including orienting towards the female, tapping, wing vibration, licking and attempted copulation [18]. The repeat training courtship suppression assay tests an individual male's ability to remember that his advances have been previously rejected by a female. Once a female Drosophila has mated, she will rebuff the advances of male flies for an extended period of time. A virgin male fly will court vigorously when presented with a female, however his behavior can be modified by her continued rejection, such that when presented with a second mated female or an immobilized virgin, his effort at courtship are reduced. This phenomenon is termed courtship suppression and is a reliable and easily tested model for both short and long-term memory [19], [20], [21], [22], [23].

A one-hour training session has been shown to produce a strong short-term memory that decays after two to three hours when tested with an anaesthetized virgin [24] and by eight hours when tested with a mobile mated female [19]. Memory that lasts longer than 30 minutes after a one hour training session is dependent on an intact mushroom body [22], however an earlier phase of courtship memory (0–30 mins) is thought to be independent of the mushroom body, as mushroom body-ablated flies display normal immediate recall after a one hour training session [22]. During a training session of five to eight hours duration, a male will engage in multiple bouts of courtship with a mated female with breaks in between. These repeated attempts at mating are thought to represent the repetition that is required for consolidation of some types of LTM [25], [26] and result in formation of a robust LTM [19], [22], [27], [28], [29] that has been shown to persist at least five to seven days after training [22], [28], [29].

Long-term courtship memory also is dependent an intact MB [22] and a functional cAMP signaling cascade as several mutants with defects in the cAMP cascade, namely *rutabaga (rut178), dunce (dnc1), DCO (DCOB3)* and *amnesiac (amn28A)*, display impaired LTM five days after a seven hour training session [29]. Moreover, long-term courtship memory is blocked by overexpression of dCREB2-b, a repressor isoform of CREB but one-hour memory is unaffected [30].

The importance of HDACs for Drosophila memory has not been previously reported. HDACs are highly conserved across species and the Drosophila genome contains five zinc-dependent HDACs: Rpd3, HDAC3, HDAC4, HDAC6 and HDAC11 [31]. Rpd3 is a Class I histone deacetylase, categorized by its similarity to yeast Rpd3, and it shares approximately equal homology to human HDAC1 and HDAC2 [32]. Rpd3 mRNA is abundant in the adult fly brain [33], suggesting a post-mitotic function. Here, we examined the reliance of long-term courtship memory on Rpd3. We found that while modulation of Rpd3 levels with the predominantly mushroom body specific driver OK107 had no observed impact on immediate recall or one-hour memory, 24-hour LTM was severely impaired. Surprisingly, both overexpression as well as RNAi-mediated knockdown of Rpd3 impaired long-term courtship memory, suggesting that the dose of Rpd3 is critical for normal LTM.

## Results

### Rpd3 is expressed throughout the fly brain

**Figure 1:**
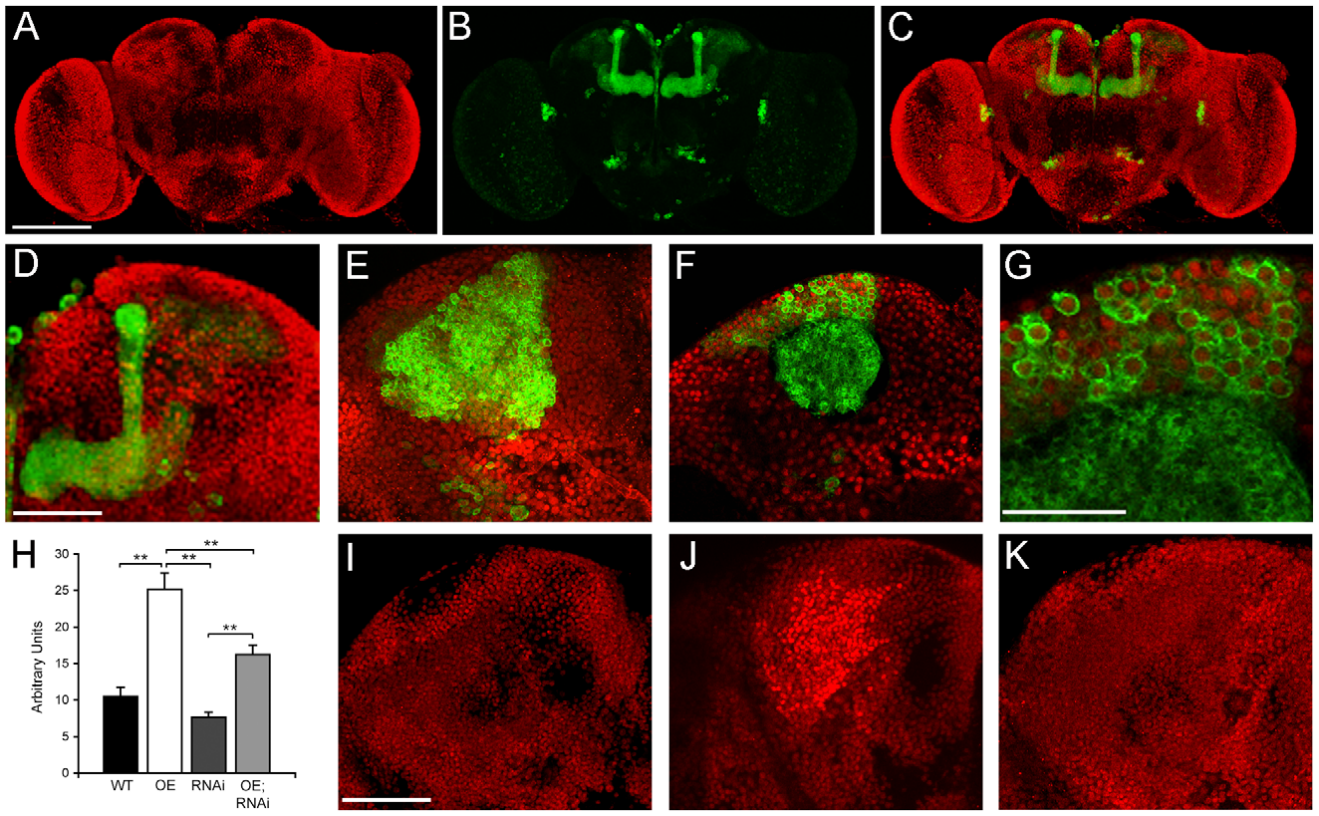
Rpd3 expression in the brain. A–G. Immunohistochemistry with an anti-Drosophila Rpd3 antibody on whole mount brains expressing CD8::GFP driven by OK107-GAL4. A. Frontal confocal projection through the brain showing widespread Rpd3 expression in neuronal nuclei. B–C. CD8::GFP is expressed predominantly in the mushroom body, with additional expression at a lower level in cell bodies of the optic lobes, pars intercerebralis and suboesophageal ganglion. D. Magnification of one hemisphere of the mushroom body shown in C. E. Posterior confocal projection through one hemisphere of the mushroom body (similar region to that of D but from a posterior orientation) which shows ubiquitous expression of Rpd3 in nuclei and Kenyon cells that are genetically labeled by CD8::GFP. F. A single confocal plane (1 µm) through the mushroom body at the level of the calyx (approximately center of the Z stack) shows Rpd3 is expressed in Kenyon cell nuclei with a halo of GFP expression in the cell membrane and calyx (dendritic processes). G. Magnification of F. Further images of Rpd3 and CD8::GFP expression in the brain and images showing colocalization of Rpd3 with ELAV can be found in Fig S1. H. Quantification of Rpd3 expression in Kenyon cells. Brains were dissected from flies that were subjected to genetic manipulation of Rpd3 levels via OK107-GAL4 driven expression of an Rpd3 RNAi hairpin, an Rpd3 cDNA, or harbouring both the Rpd3 hairpin and Rpd3 cDNA. Images of brains immunostained with Rpd3 were collected with an Olympus DP70 camera and expression was quantified in Image J (n≥10 group). Statistical significance was assessed by one-way ANOVA with post-hoc Tukey's HSD test (**p<0.01). I–K. Confocal projections of the Kenyon cells (same region as E) provide representative images of control Rpd3 expression (I), Rpd3 overexpression (J) and Rpd3 knockdown (K). The spatial location of Kenyon cells expressing the RNAi hairpin and Rpd3 cDNA is similar to that of CD8::GFP in (E). Abbbreviations: CON (control GAL80ts/+; OK107/+); OE (GAL80ts/+; Rpd3OE/+; OK107/+); RNAi (Rpd3RNAi/GAL80ts; OK107/+); OE;RNAi (Rpd3RNAi/GAL80ts; Rpd3OE/+; OK107/+); . Scale bars: A, 100 µm (for A–C); D, 50 µm (for D–F); G, 25 µm; I, 50 µm (for I–K).

Rpd3 expression in the fly brain was characterized by immunohistochemistry on whole mount adult brains with an anti-Drosophila Rpd3 antibody that recognizes an 18 amino acid sequence at the C-terminus of Drosophila Rpd3, but not other HDACs [34]. We found that Rpd3 was expressed ubiquitously throughout the brain in neuronal nuclei (Fig. 1A), and co-localized in almost all cells with the pan-neuronal ELAV protein (Fig S1Q–W). In order to visualize the structure of the mushroom bodies with respect to Rpd3 expression and to determine which of these cells are genetically labeled by the GAL4 driver OK107 [35], immunohistochemistry for Rpd3 was performed on brains expressing CD8::GFP under control of OK107-GAL4 (Fig. 1A–G). We selected this driver for its ability to facilitate high expression in Kenyon cells, the intrinsic neurons of the mushroom body. We used the reporter CD8::GFP as it is targeted to the plasma membrane, thus labeling neuronal processes and outlining the GFP void nucleus. Similarly to previous reports [16], we observed that OK107-GAL4 drives expression predominantly in the mushroom body, but is also expressed at lower levels elsewhere in the brain, including the pars intercerebralis, suboesophageal ganglion and optic lobes (Fig. 1B). In the Kenyon cells, CD8::GFP expression is observed in the cytoplasm surrounding the Rpd3 positive nuclei (Fig. 1F and G) and Rpd3 was detected in all cells that expressed CD8::GFP. Serial frontal images of whole mount brain show Rpd3 and CD8::GFP expression in a more detailed manner Fig S1A–P).

### Knockdown of Rpd3 impairs LTM

To examine the requirement of Rpd3 for normal LTM, we used a fly strain harboring a UAS-Rpd3 RNAi construct (Rpd3RNAi) for GAL4-mediated knockdown of Rpd3. To provide an initial assessment of the efficacy of the RNAi, we crossed Rpd3RNAi homozygotes to tubP-GAL4/TM3,Sb flies that ubiquitously express GAL4 through development. Homozygous Rpd3 mutants are non-viable [36], thus with sufficient knockdown the RNAi phenotype should also be lethal. No flies that carried both Rpd3RNAi and tubP-GAL4 survived to adulthood, indicating substantial knockdown of Rpd3 had occurred (237 Rpd3/TM3,Sb vs zero Rpd3RNAi/tubP-GAL4 siblings).

To quantify the extent of Rpd3 knockdown, we compared Rpd3 expression in brains of flies expressing Rpd3RNAi under control of OK107-GAL4 with that of controls (OK107/+). Brains were examined with a stereo fluorescence microscope, images captured and analyzed using software that can quantify color intensity of a selected region. We have previously used this approach to quantify expression levels of fluorescent proteins in the eye and found the results were comparable to those obtained by measuring fluorescence intensities of soluble head extracts in a fluorometer [37]. We also measured Rpd3 expression in flies that overexpress the full length Rpd3 cDNA (Rpd3OE), as well as in flies expressing both Rpd3RNAi and Rpd3OE (Fig. 1H). The level of knockdown was relatively modest and did not quite reach significance (p = 0.06), which may be due at least in part to variability in staining between brains. In Rpd3OE brains, Rpd3 overexpression was approximately 2.5-fold higher than controls (**p<0.01). Combination of this construct with of Rpd3RNAi decreased Rpd3 expression significantly compared to Rpd3OE alone (**p<0.01). Representative confocal projections through the mushroom body are shown in Fig. 1I–K. The spatial pattern of Rpd3 knockdown (1I) and overexpression (1J) is very similar to that of cells expressing CD8::GFP (1E), which is used to visualize the Kenyon cells that are genetically labeled by OK107.

In order to assess whether Rpd3 is involved in LTM, we used OK107-GAL4 to drive RNAi-mediated knockdown of Rpd3. The temporal and regional gene expression targeting (TARGET) system [15] was also included in order restrict Rpd3 knockdown to adult brains. In this system, flies are raised at 19°C, at which temperature GAL4-mediated gene expression is inhibited by GAL80ts. When the temperature is raised to 29°C, GAL80ts is inactivated and transgene (i.e. RNAi) expression ensues.

**Figure 2:**
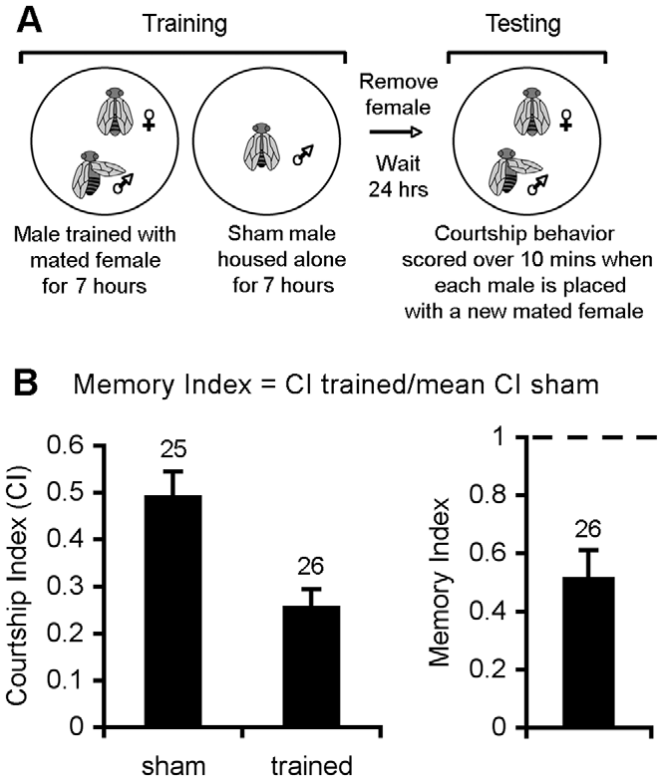
Courtship suppression assay. A. Courtship Protocol. For training, a female who was mated the previous night and a virgin male are placed together in a mating chamber. The male is allowed to court for seven hours, after which time the female is removed. At the same time, a naïve “sham” male is housed alone. To measure LTM, 24 hours later each male is placed with another freshly mated female and his courtship behavior is scored for 10 minutes. This provides a courtship index, the percentage of time he spends courting. B. Calculation of the memory index (MI). In order to compare memory between genotypes, an MI is calculated, which is the mean of CI of each trained fly divided by the mean CI of the sham group, i.e. MI =  CI_trained_/mCI_sham_. An MI of 0 is the highest possible memory score and an MI of 1 indicates that memory is the same as that of sham controls. A trained male with normal LTM will typically court about 40–60% less than a naïve male, which in this example for wild type males, corresponds to an MI of 0.51. The number of trained males tested (n) is shown above the bar. In all cases, the number of sham males tested was ±1 of the number of trained males tested.

The repeat training courtship assay was used to assess 24 hour LTM (described in Fig. 2).

In this assay, wild-type flies subjected to a seven hour training session form a robust long-term memory that is stable for at least 24 hours [19]. Memory was compared between groups by calculation of a memory index (MI), which is calculated by dividing the courtship index (CI) of each test fly by the mean CI of the sham flies (CI_trained_/mCI_sham_). This analysis allows comparison of memory between genotypes [20], [21], [23]. Memory is measured on a scale of 0 to 1, with 0 the highest memory performance possible, and a score of 1 indicating that memory is the same as that of sham controls.

**Figure 3:**
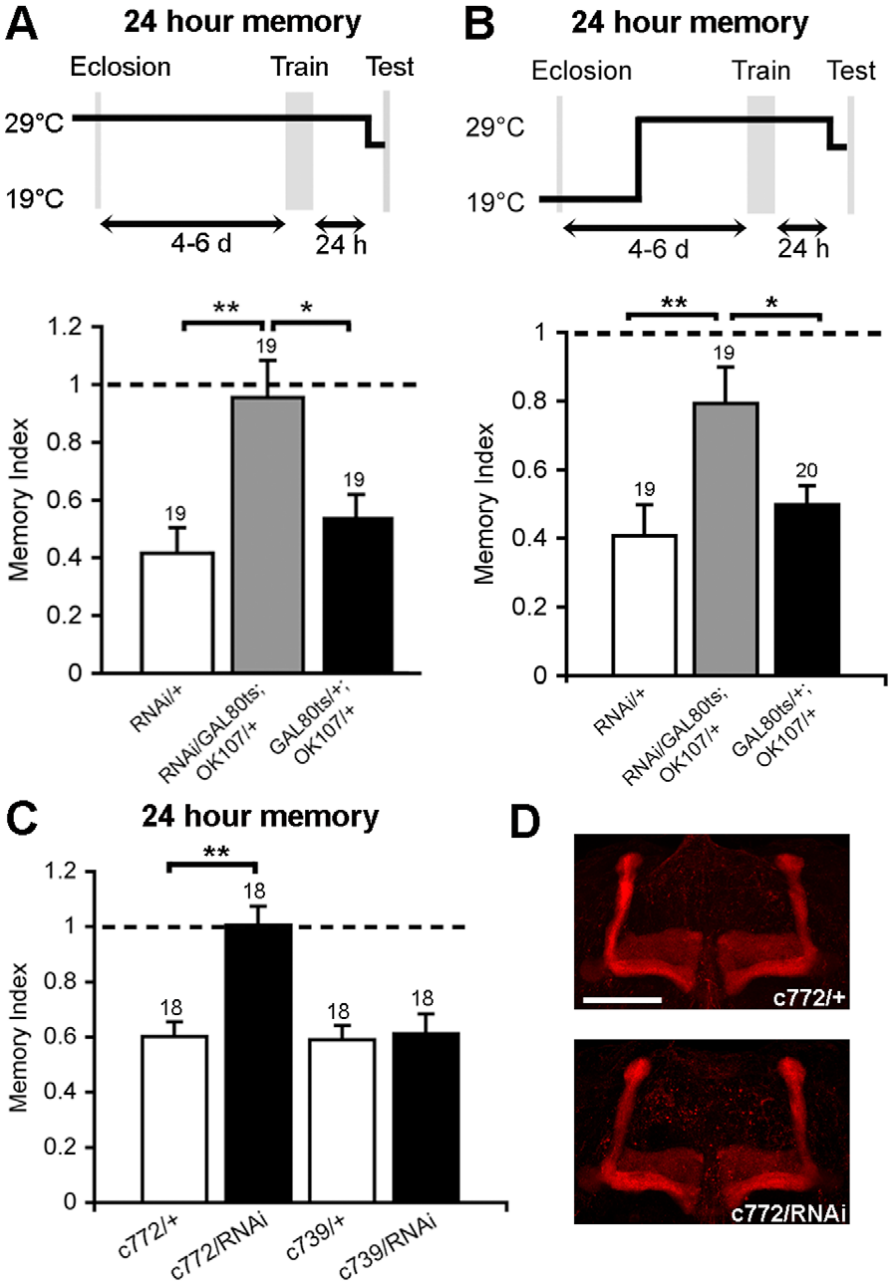
OK107 and c772-mediated knockdown of Rpd3 impairs LTM. A. Flies were reared and trained at 29°C then transferred to 25°C thirty minutes prior to testing, which was also at 25°C. Raising and training flies at 29°C did not impair LTM in parental control flies, confirming that the higher temperature itself was not detrimental to LTM formation. Knockdown of Rpd3 throughout development with OK107-GAL4 resulted in a significant impairment in LTM compared to control genotypes (*p<0.05, **p<0.01) B. Flies were raised to adulthood at 19°C and switched to 29°C three days prior to testing at 25°C. The loss of LTM in the Rpd3 knockdown males demonstrates that the memory phenotype was caused by an adult-specific decrease in Rpd3 (*p<0.05, **p<0.01). C. c772-GAL4 mediated knockdown of Rpd3 abolished LTM whereas the GAL4 driver control flies had normal LTM (**p<0.01). Knockdown of Rpd3 with c739-GAL4 did not affect LTM. D. Confocal projection through the mushroom body lobes stained with FasII reveal no obvious abnormalities in lobe structure caused by c772-GAL4 knockdown of Rpd3. Scale bar = 50 µm.

To confirm that the high temperature used for GAL80ts control does itself not affect memory formation or storage, we firstly raised and trained the flies at 29°C prior to testing. We found that both of the parental controls showed robust LTM, confirming that LTM was not adversely affected by high temperature (Fig. 3A). Strikingly, knockdown of Rpd3 resulted in a complete abolishment of LTM. Thus, it appeared that Rpd3 was required for consolidation, storage and/or retrieval of LTM. However, it was possible that normal Rpd3 expression was simply required for correct neuronal development. To address this issue, we then restricted the knockdown of Rpd3 to adulthood by raising the flies at 19°C and then switching them to 29°C after eclosion (three days prior to training), in order to avoid any potential developmental effects. This also resulted in a decreased capacity for LTM (Fig. 3B), providing evidence that post-mitotic activity of Rpd3 is important for normal LTM.

Although the highest expression driven by OK107-GAL4 is in the mushroom body, it does drive lower expression in other regions of the brain [16]. To further examine which part(s) of brain is/are dependent on wild-type levels of Rpd3 for LTM formation, we crossed the Rpd3RNAi line with two other MB GAL4 drivers, c772 [38], [39] and c739 [39]. Like OK107, c772 is expressed throughout the mushroom body, whereas c739 is expressed in the α/β lobes only. Both drivers are also expressed in a very similar pattern to OK107 in the rest of brain [16]. Knockdown of Rpd3 with c772 resulted in a significant deficit in LTM compared to the driver only control (Fig. 3C, **p<0.01). This was not due to gross abnormalities in mushroom body formation, as FasII staining did not reveal any obvious alterations in architecture of the mushroom body (Fig. 3D). However, c739-driven expression of Rpd3RNAi had no impact on LTM. (Fig. 3C).

**Figure 4:**
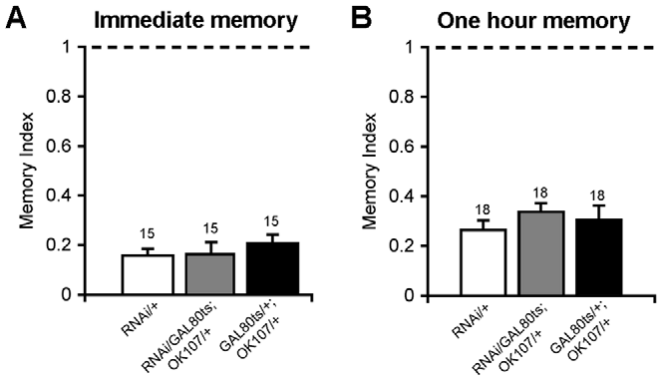
Knockdown of Rpd3 has no impact on STM. To test the effect of OK107-GAL4 knockdown of Rpd3 on STM, flies were raised to adulthood at 19°C and switched to 29°C three days prior to testing. For evaluation of immediate recall (A), flies were trained for one hour and then tested, and for STM (B) flies were tested one hour after training. All genotypes showed normal immediate recall and one hour STM, with no difference between Rpd3 knockdown flies and parental controls.

We next sought to investigate the impact of Rpd3 knockdown on early phases of courtship memory. If the action of Rpd3 on memory is occurring through its histone deacetylase activity, thereby effecting changes in gene expression, then we anticipated that OK107-mediated Rpd3 knockdown would not affect STM. To that end, we subjected males to a one-hour training session and then tested either immediately or one hour post-training. In all genotypes there were no differences in STM between Rpd3 knockdowns and controls at either time point (Fig. 4A,B).

### Inhibition of HDAC activity impairs LTM

**Figure 5:**
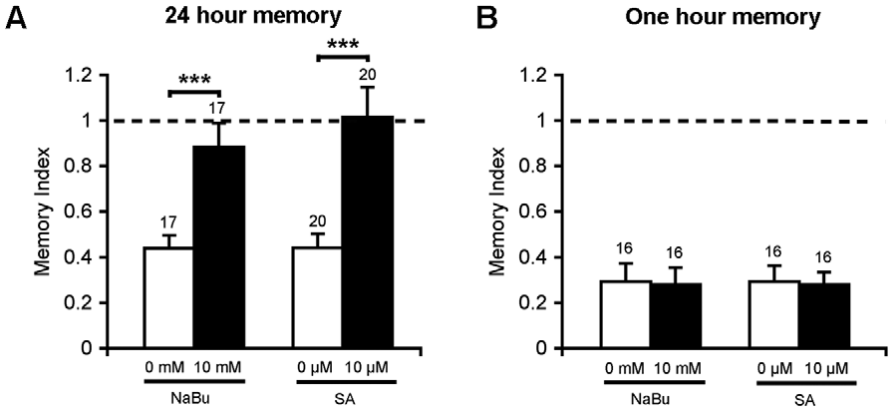
HDAC inhibition impairs LTM but not STM. A. Canton S males were treated with NaBu or SA for 24 hours prior to training and trained in chambers containing NaBu or SA in the diet. Naïve courtship activity was unaffected by NaBu (0 mM NaBu CI = 0.44 vs 10 mM NaBu CI = 0.49, p = 0.469, Mann Whitney *U* test), but NaBu treated males showed impaired LTM compared to vehicle treated controls (***p<0.001). SA treated males also showed no LTM compared to the corresponding control males (***p<0.001, Mann Whitney *U* test), with no significant effect on naïve courtship levels (0 mM SA CI = 0.48 vs 10 mM SA CI = 0.39, p = 0.062). B. One hour STM was not affected by either NaBu or SA treatment.

The impact of a reduction of Rpd3 levels on courtship LTM is intriguing, given that inhibition of HDAC activity has been correlated with improved memory in other animals. One explanation for these data is that Rpd3 may be exerting its effects on LTM through mechanisms independent of histone deacetylation, such as protein-protein interactions. To test this theory, we examined the effect of HDAC inhibition on LTM. Canton S males were treated with the short chain fatty acid HDAC inhibitor NaBu for 24 hours until immediately prior to testing. Naïve courtship activity was not affected by NaBu, however no LTM was formed (Fig. 5A). To confirm that this effect was not specific to NaBu, we repeated the experiment using 6-(1,3-Dioxo-1H, 3H-benzo[de]isoquinolin-2-yl)-hexanoic acid hydroxyamide (Scriptaid, Sigma-Aldrich, henceforth referred to as SA), a hydroxamic acid containing HDAC inhibitor that acts at a 1,000-fold lower concentration than NaBu. SA treatment resulted in a complete abolishment of LTM (Fig. 5A), with no significant effect on naïve courtship levels. STM was unaffected by either SA or NaBu (Fig. 5B).

### Overexpression of Rpd3 impairs LTM

**Figure 6:**
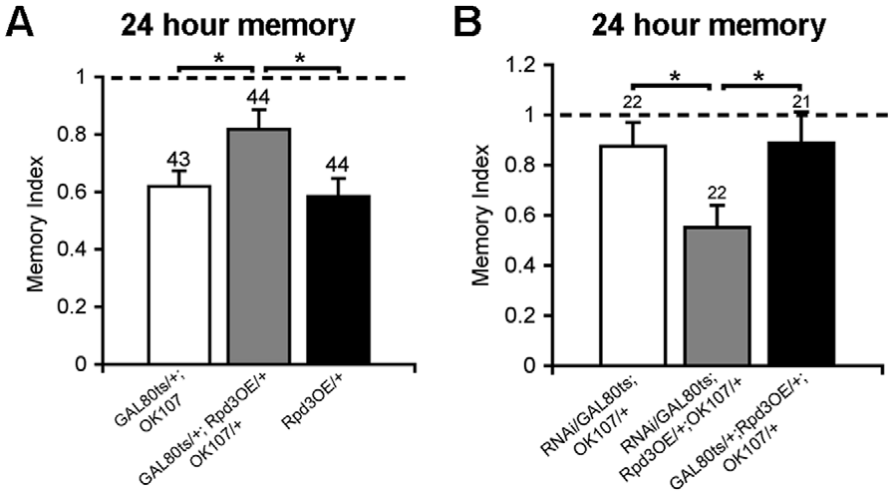
Overexpression of Rpd3 and rescue of the LTM deficit. A. Adult males in which Rpd3 was overexpressed via OK107-GAL4 showed a significant impairment in 24 hour LTM compared to parental controls (*p<0.05) B. In comparison to the LTM deficit caused by either overexpression or knockdown of Rpd3, LTM was restored in male flies that expressed both Rpd3RNAi and Rpd3OE (*p<0.05).

The phenotype observed after knockdown of Rpd3 led us to examine the effect of overexpression of Rpd3 on LTM. We generated transgenic flies containing the UAS sequence fused to a full length Rpd3 cDNA (Rpd3OE) and induced OK107-mediated expression of Rpd3OE via GAL80ts. On assessment of 24 hr LTM we found that increasing the level of Rpd3 also resulted in a specific impairment of LTM (Fig. 6A).

### Co-expression of Rpd3OE and Rpd3RNAi restores LTM

To determine whether restoration of Rpd3 levels via co-expression of Rpd3OE and Rpd3RNAi would rescue the memory phenotype, we tested courtship LTM in flies expressing both Rpd3RNAi or Rpd3OE alongside flies expressing the individual constructs. As previously observed, OK107-mediated expression of the individual knockdown and overexpression constructs impaired LTM, however in flies expressing both Rpd3RNAi and Rpd3OE, the memory phenotype was restored, with a memory index indiscernible from wild type (Fig. 6B).

### Co-expression of Rpd3OE and Rpd3RNAi rescues the lethal tubP-GAL4 phenotype

We were also interested in testing whether overexpression of Rpd3 could rescue the lethality caused by tubP-GAL4-mediated expression of Rpd3RNAi. At 25°C, expression of Rpd3OE was lethal, indicating that not only a reduction but also an excess of Rpd3 perturbs normal development (Table 1). However combination of both Rpd3OE and Rpd3RNAi partially rescued the lethal phenotype to 18.4% survival, and the survival rate rose to 59.2% when the flies were raised at 18°C. It is not immediately clear why raising the flies at 18°C increased viability but we hypothesize that it is related to GAL4 activity increasing with temperature [40]. The efficiency of Rpd3 knockdown was somewhat modest compared to the approximately 2.5-fold increase in Rpd3 when overexpressed (based on OK107-driven expression in the brain). The level of overexpression was sufficiently high such that in the presence of the RNAi, Rpd3 protein was not decreased to a wild type level. Given that GAL4 is more active at 25°C than 18°C [40] we surmise that the increased survival at 18°C is likely reflective of the net level of Rpd3 being brought closer to the wild-type level.

**Table 1:** Survival of flies following tubP-GAL4-mediated expression of UAS-HDAC constructs.

Strain crossed to tubP-GAL4/TM3, Sb	Temp (°C)	Sb males	WT males	Sb females	WT females	Total survival of WT (%)
W(CS10)	25	317	346	318	357	111
Rpd3RNAi	25	264	0	288	0	0
Rpd3OE	25	456	0	470	0	0
Rpd3RNAi;Rpd3OE	25	436	71	352	74	**18.4**
Rpd3RNAi	18	211	0	234	0	0
Rpd3OE	18	360	0	348	0	0
Rpd3RNAi;Rpd3OE	18	256	160	224	124	**59.2**
HDAC3RNAi	25	268	0	271	0	0
HDAC3RNAi;Rpd3OE	25	364	0	347	0	0

Each UAS bearing fly strain was crossed to *P*{*tubP–GAL4}LL7/TM3, Sb* and the number of Sb and non Sb adult progeny counted for each cross. Sb, stubble bristles.

The Drosophila genome contains two Class I HDACs, Rpd3 and HDAC3. To examine if there is redundancy between them, the effects of tubP-GAL4 mediated expression of HDAC3 RNAi (VDRC) on lethality was examined. HDAC3 RNAi was also lethal and co-expression of Rpd3OE did not compensate for the reduction in deacetylation, suggesting that the two HDACs have some non-overlapping targets.

## Discussion

Transcription of genes involved in synaptic plasticity is tightly regulated [41] and it is becoming increasingly evident that key regulators in this process are HDACs and HATs, with interventions that change HAT or HDAC activity resulting in altered LTM formation in various animal models of LTM [1], [3], [7], [8]. Here, we provide evidence that the Class I HDAC Rpd3 plays an integral role in memory processes in Drosophila.

Knockdown of Rpd3 via the GAL4 drivers OK107 and c772, which drive expression in all lobes of the mushroom body, resulted in deficits in long-term courtship memory. LTM was not perturbed by Rpd3 knockdown via the α/β lobe driver c739. Given that OK107 and c772 both drive highest expression in the MB, and display similar extra-mushroom body expression (in regions including the optic lobes and suboesophageal ganglion), to c739 [16], combined with evidence that the mushroom body is a brain area critical for long-term courtship memory [22], it appears likely that knockdown of Rpd3 in the mushroom body is responsible for the memory phenotype. In addition, the lack of a memory deficit with c739 suggests that wild type levels of Rpd3 in the α/β lobes are not required for LTM, and the memory effect of Rpd3 may be mediated through the α′/β′ and/or γ lobes. It is, however, important to consider that drivers for a particular lobe are not always equally expressed within a Kenyon cell subtype - the expression pattern of c739 has been shown to complement that of c772 in the α/β lobes, with c772 expression in the peripheral neurons and c739 expressed in the internal neurons [39], [42]. Therefore it is possible that Rpd3 in the peripheral α/β neurons may play a role in courtship LTM. Further experiments will been needed to confirm the involvement of specific Kenyon cell subtypes and to examine potential contributing effects of other regions of the brain.

Overexpression of Rpd3 also resulted in impairment of long-term courtship memory, and LTM was rescued by RNAi-mediated knockdown of Rpd3 in the same cells. From these data we infer that the excess deacetylase activity resulting from Rpd3 overexpression inhibits the plasticity-related gene transcription that is required for LTM formation. Overexpression of HDAC2, a human counterpart of Rpd3, in the mouse brain also causes a deficit in long-term memory [13] and ChIP analysis has been used to identify that HDAC2 is enriched at the promoters of many genes involved in synaptic plasticity and growth [13]. It should be noted that knockout of HDAC2 was not restricted to adulthood, therefore there is a possibility that developmental or compensatory effects could have also contributed to the memory phenotype.

More studies are required to dissect the precise mechanisms by which HDACs regulate LTM, including the exact timing requirement and dynamics of HDAC activity as well as identification of critical interacting partners and their effects on changes in gene expression. However a working model, elegantly described as the “molecular brake pad hypothesis” [43] proposes that transcription complexes containing HDACs are present at plasticity-related genes and keep their expression in check until synaptic activity-dependent release of the complexes occurs, allowing access of HATs and transcription factors. We hypothesize that in this study, the LTM deficit caused by overexpression of Rpd3 results from excess Rpd3 “putting the brakes” on activity-dependent transcription required for courtship LTM.

Intriguingly, RNAi-mediated knockdown of Rpd3 caused impairment of courtship LTM while leaving normal courtship activity and STM intact. This is in contrast to results described in mouse models where a reduction in HDAC activity enhances memory [13], [14]. In effect, removing the brake pad led to a paradoxical deficit in LTM. Restoration of Rpd3 via overexpression of wild-type Rpd3 completely rescued the memory phenotype, indicating that these results are unlikely to be caused by off target effects. The identical impairment of LTM caused by pharmacological inhibition of HDAC activity suggest that the effect of Rpd3 knockdown is likely to be due to alterations in histone acetylation at specific target genes, rather than other effects such as disruption of critical protein-protein interactions. These effects are likely mediated through either or both of the Class I HDACs, Rpd3 and HDAC3, as they are by far the most sensitive HDACs to both NaBu and SA inhibitors [44].

It is becoming increasingly clear that the interplay between HATs and HDACs is complex and far from the simple notion that HATs induce and HDACs repress gene expression. Indeed, more genes are actually upregulated than downregulated on overexpression of Rpd3 in S2 cells [44] and the impact of RNAi-mediated knockdown of Rpd3 in the same cell line was an increase in transcription. Several studies have also shown that inhibition of HDAC activity does not result in a global increase in all CREB targets and indeed, HDACs can play a role in activation of gene expression. Focal administration of the HDAC inhibitor Trichostatin A into the mouse hippocampus increases histone acetylation and enhances contextual fear memory, but induces expression of only a subset of CREB target genes [45]. Moreover, induction of expression of a subset of CREB target genes in PC12 cells by a constitutively active CREB mutant could be blocked by application of TSA, indicating that HDAC activity was required for the activation of these genes [46].

In addition to the complex regulation of gene expression by HATs and HDACs, there is evidence that an increase in histone acetylation does not always correlate with increased LTM. Transgenic mice containing a deletion or truncated form of the HAT p300 show decreased histone acetylation and impairments in hippocampus-dependent memory function [47], [48]. However, on the contrary, specific knockout of p300 in the infralimbic region of the mouse brain enhances fear extinction memory and also facilitates long term potentiation (LTP) [49]. These data show that in different contexts, the activity of this HAT can both enhance or constrain LTM.

Here, in the first examination of HDAC function in Drosophila memory, we found that wild-type levels of Rpd3 in regions of the brain genetically labeled by OK107 are required for normal courtship LTM. More research is required to tease out specific mechanisms that result in an impairment in LTM when Rpd3 levels are reduced. However, one explanation of our data is that a reduction in Rpd3 leads to increased expression of a repressor that regulates the plasticity-related proteins required for normal LTM. In this scenario, reduction of Rpd3 would allow increased histone acetylation and expression of plasticity-related proteins but also of a repressor or interacting protein that perturbs LTM. An alternative explanation arises from the observations from CHiP analyses that both yeast Rpd3 and its human counterpart HDAC1 bind predominantly to active genes [50], [51]. An explanation of this counterintuitive phenomenon is that promoter binding of HATs and HDACs occurs in a cyclical manner, with histone acetylation promoting initiation of transcription. HDACs then bind and remove acetyl groups, thus clearing the promoter to allow a second round of PolII recruitment [51], [52]. In yeast, the Rpd3S transcription complex is necessary for preventing spurious transcription within genes, by deacetylating the body of open reading frames [53]. Together these data describe a role for Rpd3 in gene activation, thus a decrease in the amount of Rpd3 may perturb normal gene expression in some circumstances. Future examination of the gene targets of Rpd3 and the transcriptional profiles resulting from Rpd3 overexpression and knockdown in the adult brain will shed light on the specific mechanisms by which Rpd3 regulates courtship LTM in Drosophila.

## Materials and Methods

### Fly Strains

All flies were cultured on standard medium on a 12 hour light/dark cycle and maintained at a temperature of 25°C unless otherwise indicated. Canton S flies were used as wildtype controls. *w^1118^; P{GD4513}v30600* (Rpd3RNAi) and *w^1118^; P{GD9732}v20814* (HDAC3-RNAi) strains were obtained from the Vienna Drosophila RNAi Center and the genotypes of the stocks were confirmed according to the transgene verification protocol from the VDRC, i.e. amplification of a product of expected size following PCR of genomic DNA with a forward primer (designed to the promoter) and a reverse primer identical to the EcoRI primer that was used for cloning the RNAi. w*; P{w+mW.hs = GawB}OK107 (OK107-GAL4), *y1w67c23; P{w+mW.hs = GawB}c739* (c739-GAL4), and y^1^ w*; P{tubP–GAL4}LL7/TM3, Sb^1^ (tubP-GAL4) were obtained from the Bloomington Drosophila Stock Center. *P[GAL4] c772* (c772-GAL4), w*; P{w+mC = tubP-GAL80ts}10 (tubP-GAL80^ts^) and *w(CS10)* strains were kindly provided by R. Davis (The Scripps Research Institute, Jupiter, FL).

To generate the UAS-Rpd3OE strain, the pOT2 plasmid containing a full length Rpd3 cDNA clone (GM14158, BDGP Gold Collection, Drosophila Genome Research Center) was digested with *Eco*RI (partial) and *Xho*I to release the full 2157 bp cDNA. This was cloned into *Eco*RI and *Xho*I of pUASTattB. The result̀ing plasmid was microinjected into embryos of the *y w, P{hs-flp}; P{3xP3-RFP = attP-86F}; P{3xP3-RFP = phic-31{3xP3-GFP = vas-phic31}}102F* strain containing attP landing site on the third chromosome. The attP strain and pUASTattB plasmid were obtained from Konrad Basler, University of Zurich, [54]. All strains were outcrossed for a minimum of five generations to *w(CS10)* flies. A homozygous line harbouring *w(CS10)*; *P{w+mC = tubP-GAL80ts}10* and *P{w+mW.hs = GawB}OK107* (tubP-GAL80_ts_;OK107-GAL4) was generated by standard genetic crosses, as was *w(CS10); P{GD4513}v30600* and *P{3xP3-RFP = attP-86F}UAS-Rpd3OE* (Rpd3RNAi; Rpd3OE). For analysis of the effect of modulation of Rpd3 levels on adult viability, the appropriate transgenic strains and *w(CS10)* were crossed to *tubP–GAL4}LL7/TM3, Sb.* The percentage survival was determined by the ratio of the surviving *Sb* and non-*Sb* adults.

Genotypes of males tested in the courtship assay were: *w(CS10); P{w+mC = tubP-GAL80ts}10/+*; *P{w+mW.hs = GawB}OK107/+* [GAL80ts/+;OK107/+]; *w(CS10); P{GD4513}v30600/+* [Rpd3RNAi/+]; *w(CS10); P{GD4513}v30600/P{w+mC = tubP-GAL80ts}10; P{w+mW.hs = GawB}OK107/+* [Rpd3RNAi/GAL80ts;OK107/+]; *w(CS10); w(CS10); p[GAL4]c772/P{GD4513}v30600/+* [Rpd3RNAi/c772]; *p[GAL4]c772/+* [c772/+]; *y1w67c23; P{w+mW.hs = GawB}c739/P{GD4513}v30600* [c739/Rpd3RNAi]; *P{w+mW.hs = GawB}c739/+* [c739/+]; *P{3xP3-RFP = attP-86F}UAS-Rpd3OE/+* [Rpd3OE/+]; *w(CS10); P{GD4513}v30600/+ P{3xP3-RFP = attP-86F}UAS-Rpd3OE/+; P{w+mW.hs = GawB}OK107/+* [Rpd3RNAi/GAL80ts; Rpd3OE; OK107/+]. All of these genotypes harboured transgenic constructs containing the mini-white gene in a *w(CS10)* background and had an eye colour that appeared wild type.

### Behavioral Analyses

The repeat training courtship assay [19], [22], [24] was used to assess memory. The premise of this assay is that male flies learn that they have been previously rejected by a female and thus when tested with a new female, they display a decrease in courtship behavior compared to naive males. Virgin Canton S females (4–6 days old) were mated overnight with virgin Canton S males (4–6 days old) to obtain a stock of newly mated females for training. Each training chamber was a constructed from a block of transparent acrylic containing a hole measuring 15 mm in diameter by 15 mm in depth. Food was poured into the chamber up to a level of 2 mm from the top and covered with a sliding acrylic lid once set. The following day, single virgin males of each genotype to be tested were aspirated into individual training chambers. A mated female was aspirated into half of the chambers (trained group), and then other half of the male group was housed alone (naïve group). To allow the memory consolidation required for LTM, flies were incubated in the training chamber for seven hours, during which multiple rounds of courting were observed in the trained group. The female fly was then aspirated from the training chamber and the males were left in their chambers for the 24 hours prior to testing. For STM, the flies were trained for one hour, then tested either immediately, or one hour after the female was removed.

The testing chamber was identical to the training chamber, except it had a depth of 3 mm and no food. Between each test, chambers were rinsed with 95% ethanol and allowed to dry. Each trained or naïve male fly was aspirated into a testing chamber containing a mated wild type female and was scored for the time spent performing stereotypic courtship behaviors (orienting towards the female, wing extension and vibration, chasing, orienting towards the female, licking) over the ten minute period. A courtship index (CI) was calculated as the percentage of the ten-minute period spent in courtship behavior. In order to compare memory across genotypes, a memory index (MI) was calculated by dividing the courtship index (CI) of each test fly by the mean CI of the sham flies of that genotype (CI_test_/mCI_sham_) [20], [21], [23]. Memory is measured on a scale of 0 to 1, with 0 the highest memory performance possible, and a score of 1 indicating that memory is the same as that of sham controls. For statistical analyses, data was arcsine transformed in order to approximate a normal distribution and significance was assessed by one way ANOVA with post-hoc Tukey's HSD test. When comparing only two genotypes (HDAC inhibitor experiments), the Mann-Whitney *U* test was used.

Male flies to be tested were collected and housed in single vials for 4–6 days. For each experiment, males of each parental genotype were tested alongside those expressing the knockdown or overexpression construct. In all experiments, the scorer was blinded to the genotype of the flies. All naïve and trained groups contained (n = 18 to 44) males except for the short-term memory and HDAC inhibitor assays (n = 15 to 20). All experiments were performed under ambient light.

For experiments using the TARGET system [15], the temperature was modulated by placing flies at the permissive temperature of 19°C (GAL80ts active) or the restrictive temperature of 29°C (GAL80ts inactive), as appropriate. For induction of transgene expression, flies were transferred to 29°C three days prior to training to allow maximum GAL4-mediated expression of the UAS construct. Flies were trained at 29°C in an incubator under white light and remained at 29°C until 30 minutes before testing, at which time they were transferred to 25°C for equilibration to the testing conditions.

The HDAC inhibitor sodium butyrate (NaBu, Sigma Aldrich) was dissolved in H_2_O to a concentration of 1 M and was diluted in molten fly culture media to a final concentration of 10 mM. Flies to be treated with NaBu were transferred onto vials containing NaBu for 24 hours prior to training and were trained in chambers containing 10 mM NaBu in the food. Scriptaid (SA, Sigma Aldrich) was diluted in DMSO to 16 mM. To produce a working solution, the stock was diluted to 10 µM in 2% sucrose. 1 ml of working solution was pipetted onto a kimwipe placed in a vial. Flies to be treated with SA were transferred onto SA containing vials for 24 hours prior to training and trained in chambers containing 10 µM SA in the food. Corresponding control flies were treated identically to the NaBu and SA flies with stock solutions made with water and DMSO, respectively, in place of the drug.

### Immunohistochemistry

Whole flies were fixed in PFAT/DMSO (4% paraformaldehyde in 1× PBS +0.1% Triton X-100+5% DMSO) for one hour then washed in 1×PBS. Brains were microdissected in 1×PBS then post fixed in PFAT/DMSO for 20 mins and stored in MeOH at −20°C. Following rehydration in PBT (1×PBS+0.5% triton X-100) brains were blocked in immunobuffer (5% normal goat serum in PBT). Brains were incubated with primary antibody (mouse anti-ELAV, 1∶100; mouse anti-FasII 1∶200 or rabbit anti-Drosophila Rpd3 (Abcam ab1767, 1∶1000) then incubated with secondary antibody (goat anti-mouse Cy3, Jackson ImmunoResearch, 1∶200 or goat–anti-rabbit Alexa 555, Molecular Probes, 1∶200) and mounted with Antifade. The monoclonal antibodies anti-fasciclin II (1D4, developed by C. Goodman) and anti-ELAV (9F8A9, developed by G.M. Rubin) were obtained from the Developmental Studies Hybridoma Bank developed under the auspices of the NICHD and maintained by The University of Iowa, Department of Biology, Iowa City, IA 52242. For quantification of Rpd3 expression, fluorescence was visualized under an Olympus SZX12 zoom Stereo Microscope with an Olympus U-RFL-T mercury burner lamp. Images were captured with an Olympus DP70 digital camera and processed using Olympus DPController software. Relative Rpd3 expression in the Kenyon cells was quantified using Image J (National Institutes of Health, Bethesda, Maryland, USA). For confocal microscopy, optical sections were taken with a Leica TCS SP5 DM6000B Confocal Microscope. Image stacks were taken at intervals of 1 µm and processed with Leica Application Suite Advanced Fluorescence (LAS AF) software.

## Supporting Information

Figure S1 **Rpd3 expression in the brain.** A–P. Immunohistochemistry with an anti-Drosophila Rpd3 antibody on whole mount brains expressing CD8::GFP driven by OK107-GAL4. A–H. Optical sections through the brain from anterior to posterior showing widespread Rpd3 expression in neuronal nuclei throughout the brain. CD8::GFP expression can been seen in all the lobes, penduncle, calyx and Kenyon cells of the mushroom body, with additional expression at a lower level in cell bodies of the optic lobes, pars intercerebralis and suboesophageal ganglion. Scale bar = 100 µm. I–P. Optical sections through the brain through one hemisphere of the mushroom body from a posterior angle showing ubiquitous expression of Rpd3 in nuclei and in Kenyon cells that are genetically labeled by CD8::GFP. Scale bar = 50 µm. Q–W. Immunohistochemistry on whole mount brain with antibodies to Rpd3, in red, and ELAV, in green. Q–S. Frontal projection of a whole mount brain. Rpd3 is expressed ubiquitously in neuronal nuclei throughout the brain, colocalizing with ELAV, a pan-neuronal nuclear protein. Scale bar = 125 µm. T–V. A 10 µm optical section at the level of the Kenyon cells (approximately the same level as M and H) shows co-expression of Rpd3 and ELAV in most neuronal nuclei, but no expression in extra-nuclear regions. Scale bar = 50 µm. W. Magnification of area surrounded by the white square in V, scale bar = 12.5 µm.
(TIF)
