# Supplementary figures and images for: Genetic Modulation of Rpd3 Expression Impairs Long-Term Courtship Memory in Drosophila
Source: PLoS One. 2011 Dec 15;6(12):e29171. doi: 10.1371/journal.pone.0029171 (PMC3240647; doi:10.1371/journal.pone.0029171)

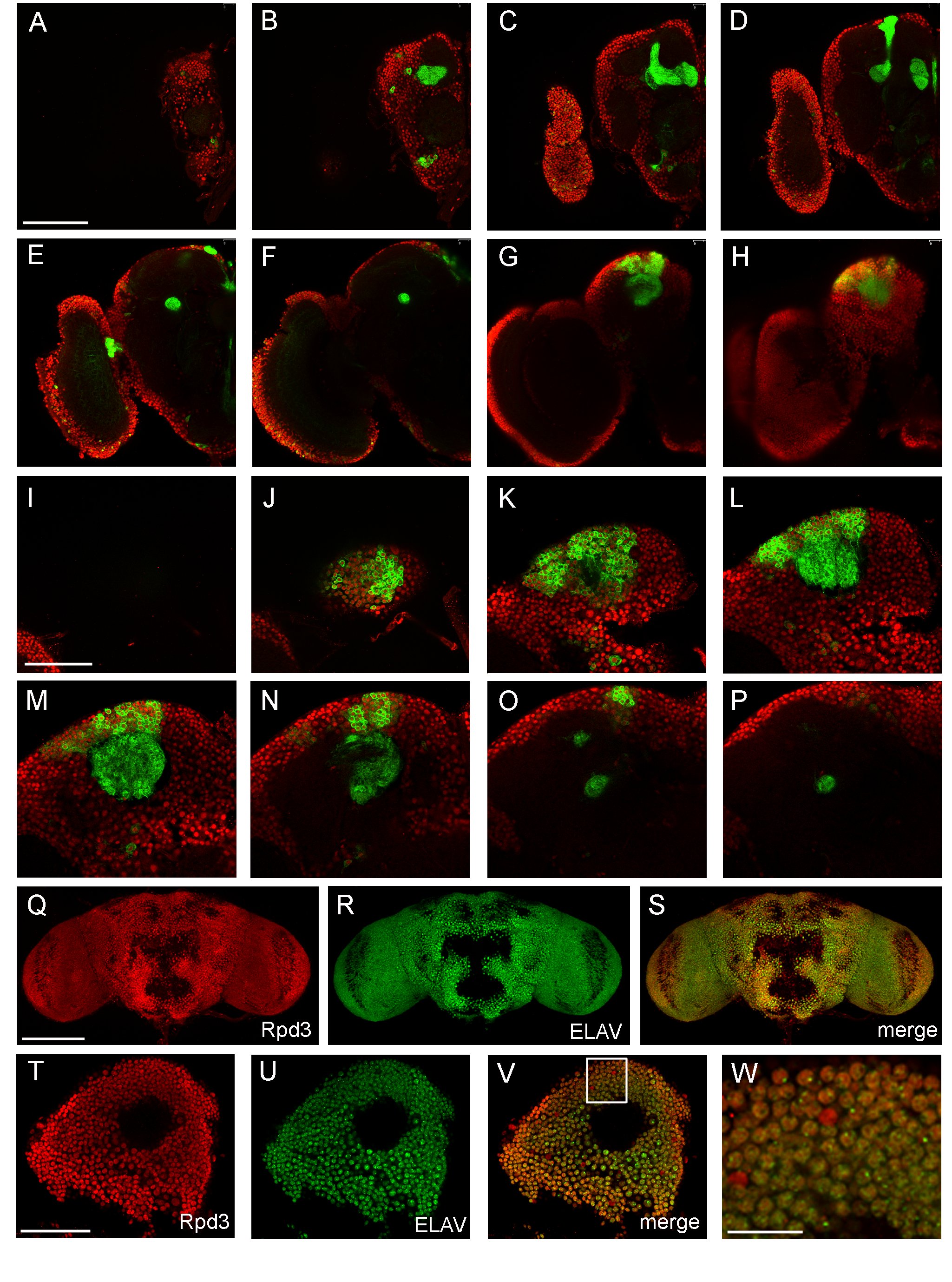

Supplement: Figure S1 — Rpd3 expression in the brain. A–P. Immunohistochemistry with an anti-Drosophila Rpd3 antibody on whole mount brains expressing CD8::GFP driven by OK107-GAL4. A–H. Optical sections through the brain from anterior to posterior showing widespread Rpd3 expression in neuronal nuclei throughout the brain. CD8::GFP expression can been seen in all the lobes, penduncle, calyx and Kenyon cells of the mushroom body, with additional expression at a lower level in cell bodies of the optic lobes, pars intercerebralis and suboesophageal ganglion. Scale bar = 100 µm. I–P. Optical sections through the brain through one hemisphere of the mushroom body from a posterior angle showing ubiquitous expression of Rpd3 in nuclei and in Kenyon cells that are genetically labeled by CD8::GFP. Scale bar = 50 µm. Q–W. Immunohistochemistry on whole mount brain with antibodies to Rpd3, in red, and ELAV, in green. Q–S. Frontal projection of a whole mount brain. Rpd3 is expressed ubiquitously in neuronal nuclei throughout the brain, colocalizing with ELAV, a pan-neuronal nuclear protein. Scale bar = 125 µm. T–V. A 10 µm optical section at the level of the Kenyon cells (approximately the same level as M and H) shows co-expression of Rpd3 and ELAV in most neuronal nuclei, but no expression in extra-nuclear regions. Scale bar = 50 µm. W. Magnification of area surrounded by the white square in V, scale bar = 12.5 µm. (TIF) [file pone.0029171.s001.tif]
